# A Contribution to the Pathology of the Vermiform Appendix

**Published:** 1894-03

**Authors:** 


*A Contribution to the Pathology of the Vermiform Appendix.* By T. N. KELYNACK, M.D. Pp. 211. London: H. K. LEWIS. 1893.

This work is based on more than 200 cases of the author's own observation, in addition to a very large number reported by other writers. A considerable amount of confusion has arisen from the use of such terms as typhlitis, and Dr. Kelynack strongly urges that a more exact nomenclature in accordance with pathological conditions is necessary.

In treating of recurrent appendicitis, there is no differentiation made between chronic relapsing appendicitis in which there is no return to absolute good health, and recurring appendicitis in which each attack may practically be regarded as an independent affection.

The chapters on symptomatology and treatment are admittedly brief, and do not add to the value of the work. After so much labour and observation as the author has expended on this subject, we naturally look for some practical results in the general principles of treatment. In this, however, we are disappointed; for it does not appear to us that either the medical or surgical treatment of this important region has been advanced by the pathological observations before us.

The book, however, contains practically all that is known of the normal and morbid anatomy of the appendix, and much trouble has been taken in the collection of statistics. At the end of the volume is a very extensive bibliography.

